# Equine metabolic syndrome in UK native ponies and cobs is highly prevalent with modifiable risk factors

**DOI:** 10.1111/evj.13378

**Published:** 2020-12-03

**Authors:** Harry B. Carslake, Gina L. Pinchbeck, Catherine M. McGowan

**Affiliations:** ^1^ Institute of Infection, Veterinary and Ecological Sciences Faculty of Health and Life Sciences University of Liverpool Leahurst UK

**Keywords:** horse, EMS, laminitis, prevalence, epidemiology, obesity

## Abstract

**Background:**

The epidemiology of equine metabolic syndrome (EMS) is poorly described.

**Objectives:**

To estimate the prevalence of EMS in native UK ponies and cobs in England and Wales and identify associated risk factors.

**Study design:**

Cross‐sectional study.

**Methods:**

Breeders registered with UK native pony breed societies and registered riding schools and livery yards within a set radius were invited to participate. All native UK ponies and cobs aged 3‐14 years and not diagnosed or being treated for conditions likely to affect insulin regulation at participating premises were eligible. Animals underwent a clinical examination and an oral glucose test while their owner or keeper completed a questionnaire by face‐to‐face interview. Data were analysed by multilevel uni‐ and multivariable modelling using insulin concentration and EMS diagnosis as outcomes.

**Results:**

A total of 354 animals were examined at 64 properties (19 studs, 19 livery yards, 26 riding schools). The overall prevalence of EMS adjusted for clustering within yard was 23.3% (95%CI 17.9%–29.8%). Risk factors associated with a diagnosis of EMS included age, being female, more sedentary main activity, obesity, and shorter periods on pasture during the summer. Compared to the Welsh section A, the other Welsh, Connemara and cob breeds all had decreased odds of EMS. Clinical manifestations of hoof growth ring and supraorbital fat scores of 3/3 were more frequent in EMS ponies and animals with a history of laminitis within the last 5 years (9.7%) were 14.4 (95% CI 5.9–35.3) times more likely to be positive for EMS than those without.

**Main limitations:**

Results may not be transferable to other breeds or age groups.

**Conclusions:**

Equine metabolic syndrome is highly prevalent in UK native ponies and cobs with modifiable risk factors including obesity and sedentary activities. Modifying risk factors could help reduce the risk of laminitis in susceptible animals.

## 1 INTRODUCTION

Equine metabolic syndrome (EMS) is a recognised collection of risk factors for endocrinopathic laminitis.^1^ Several metabolic disturbances have been associated with EMS^2^, ^3^, ^4^ but, of these, insulin dysregulation (ID) is now recognised as the defining feature of the condition.^1^

Early identification of EMS is important, as there are effective management strategies that can be implemented to reduce the likelihood of laminitis developing.^5^ This includes both identification of at‐risk animals, as well as diagnostic tests to confirm the disease. A definitive diagnosis of EMS requires demonstration of ID, for which a range of basal and dynamic diagnostic tests have been described. Dynamic tests for ID are more sensitive,^6^ but can also be more invasive and time consuming; hence less practical for use as a screening test.

Several studies have investigated the incidence of and risk factors for laminitis^3^, ^7^, ^8^ and obesity,^9^ but the epidemiology of EMS is poorly described and is generally restricted to single herds^2^, ^4^ or epidemiological studies using less sensitive tests for EMS such as basal insulin.^10^ Anecdotally, native pony and cob breeds are considered particularly susceptible to EMS, and experimental studies have shown mixed breed ponies to be more insulin resistant than Standardbreds.^11^ More recently, several metabolic traits of EMS were shown to have moderate to high heritability in Welsh ponies.^12^

Identification of horse, management and phenotypic risk factors for EMS will improve identification of at‐risk animals for further, targeted diagnostic testing, screening for ID or implementation of EMS management based on a presumptive diagnosis. Furthermore, identification of modifiable risk factors will allow more effective management strategies to be employed, ultimately reducing the risk of laminitis. Therefore, the aim of the present study was to estimate the prevalence of EMS in native UK ponies and cobs and their crosses in England and Wales, and to identify associated risk factors and clinical manifestations.

## 2 MATERIALS AND METHODS

### 2.1 Study sample

The study population included UK native ponies and their crosses and cobs aged 3‐14 years in northwest England and north Wales. Breeders registered with UK native pony breed societies and riding schools and livery yards registered with the British Horse Society (BHS, Kenilworth) or with details available on a local equine website (https://www.wirralhorse.co.uk/) and within a 50km radius of the University of Liverpool, Leahurst for breeders and livery yards, or a 75km radius for riding schools were invited by letter and email to participate in the study. Exclusion criteria were animals aged <3 or >14 years, lactating or in the last trimester of pregnancy, diagnosed with pituitary pars intermedia dysfunction (PPID) or other systemic disease, with laminitic foot pain, or receiving any medication likely to affect insulin regulation. The breed of pony was as reported by the owner. It was requested that all eligible animals at participating premises were included. Using an estimated prevalence of 25% for EMS,^10^, ^13^ a sample size of 289 animals was calculated (Epitools Epidemiological Calculators) to allow detection of an estimated prevalence of 25% with 5% precision with 95% confidence intervals. This would provide approximately 70 cases; providing 80% power to detect odds ratios of 2.5 or greater for exposures of 25% or greater in the control group.

### 2.2 Data collection

Owners were instructed to house the animals and feed only a single slice of hay (approximately 1.5 kg) on the evening before a visit.

#### 2.2.1 Clinical examination and oral glucose test

Starting between 8am and 9am, each animal underwent a brief clinical examination to establish inclusion criteria were met and to estimate bodyweight (kg), calculated from girth circumference and body length.^14^ A blood sample (*t* = 0) was collected into plain (Sarstedt, Leicester) and EDTA (Vacutainer, Becton‐Dickinson) evacuated tubes for measurement of serum insulin (insulin_0_) and plasma ACTH concentrations respectively, and for patient‐side measurement of blood glucose concentration (glucose_0_). A feed containing 1 g/kg bodyweight glucose powder (Henry Schein UK Holdings, Gillingham), mixed with 1 g/kg bodyweight low non‐structural carbohydrate chaff (Happy Hoof, Spillers, Milton Keynes) and 1ml/kg bodyweight water was given. Time to consume the feed and the weight of any residual feed after 30 minutes were recorded. Following this, a physical examination was performed by a single, experienced veterinarian blinded to questionnaire responses, which included body condition score (BSC) out of 9,^15^ cresty neck score,^16^ and semi‐quantitative grades of supraorbital fat and hoof growth‐ring morphology from 1 to 3 (Supplementary Item 1). A second blood sample was collected for measurement of serum insulin concentration (insulin_120_) and patient‐side blood glucose concentration (glucose_120_) 120 minutes after the feed. The animal was then returned to its normal management.

#### 2.2.2 Blood analysis

Following blood sampling EDTA tubes were placed immediately in crushed ice and serum tubes were allowed to clot at ambient temperature. Two to four hours following blood collection, tubes were centrifuged at 3,000 g for 10 min in a laboratory. Serum insulin concentration and, in animals aged ≥10 years, plasma ACTH concentration were measured in duplicate using chemiluminescent assays (Immulite 2000/Immulite 2000XPi, Siemens, Healthcare) previously validated in horses.^17^, ^18^ Samples with an insulin concentration greater than the upper reportable limit (300µIU/mL) were diluted with charcoal‐stripped serum.^17^ The model of the chemiluminescent analyser was updated from the Immulite 2000 to the Immulite 2000XPi after 234 animals. To adjust for the new analyser paired measurement of insulin concentration in 39 samples was performed. Regression analysis (Supplementary Item 2) was used to transform results from the Immulite 2000 to the newer Immulite 2000XPi, which are presented here. Blood glucose concentration was measured using a validated point of care glucometer (AlphaTRAK, Zoetis).^19^

#### 2.2.3 Survey data

During the visit, the owner or keeper of each horse completed a questionnaire by face‐to‐face interview by a single experienced research assistant. A range of mostly closed‐end style questions were asked examining signalment, use, exercise duration and intensity, feeding and other management practices, and medical history (Supplementary Item 3).

#### 2.2.4 Follow‐up

Following the visit, a report was sent to the horse's owner and their regular veterinary practice. For any horse testing positive for EMS or PPID advice on further diagnosis and management was enclosed.

### 2.3 Data analysis

A causal web for EMS was created (Supplementary Item 4) allowing explanatory variables to be divided into two groups: potentially causal risk factors (e.g. age, management, season of testing, Table 1), and factors considered clinical manifestations that may be a result of EMS (e.g. hoof changes, insulin_0_, history of laminitis, Table 2). Two outcomes were examined: a binary outcome of EMS diagnosis where an animal was classified as positive for EMS if insulin_0_ >33 µIU/mL, or insulin_120_ >131 µIU/mL for animals consuming 75%‐100% of feed, or >112 µIU/mL for animals consuming 50% – 75% of feed^1^ (animals consuming < 50% of feed excluded); and a continuous outcome of insulin_120_ where animals consuming <75% of feed were excluded.^20^ The continuous data were transformed using the natural logarithm of (insulin_120_ + 1), to establish a normal distribution (Shapiro‐Wilk). Animals were designated positive for PPID and thus excluded when plasma ACTH concentration was >50 pg/mL mid‐November to mid‐July and >100 pg/mL mid‐July to mid‐November.^21^ An ordinal hoof growth ring score (1‐3) was calculated as the integer of the mean of the growth ring divergence and growth ring prominence scores. The hours exercised per week walking, trotting, cantering, jumping or galloping were multiplied by 1, 2, 4, 5 or 6, respectively, and the sum calculated as an overall exercise score. Sex was categorised as male and female due to low numbers of pregnant females and stallions (Table 1).

**Table 1:** Descriptive data for potentially causative risk factors for EMS in all (n = 339), EMS positive (n = 83) and EMS negative (n = 256) ponies, univariable, multilevel logistic regression of potentially causal risk factors associated with a diagnosis of EMS (n = 339) and univariable, multilevel linear regression of potentially causal risk factors associated with Log_e_ (insulin_120_ + 1, n = 320) in native ponies and cobs in northwest England and north Wales. Categorical variables are presented as percentages, continuous variables as median (IQR)

**Variable**	**Category**	**All ponies (n = 339)**	**Lower 95% CI**	**Upper 95% CI**	**EMS positive (n = 83)**	**Lower 95% CI**	**Upper 95% CI**	**EMS negative (n = 256)**	**Lower 95% CI**	**Upper 95% CI**	**Binary outcome Odds Ratio**	**Lower 95% CI**	**Upper 95% CI**	***P*‐Value**	**Continuous outcome** **Coefficient**	**S.E.**	***P*‐Value**
**Age (years)**	n/a	9 (6 – 11)			11 (9‐13)			8 (6 – 10)			1.29	1.17	1.42	<.001	0.12	0.02	<.001
**Sex (4 categories)**	Gelding	51.6	46.3	56.9	36.1	26.6	46.9	56.6	50.5	62.6	Ref			.02	Ref		.006
	Pregnant mare	4.1	2.5	6.8	6.0	2.6	13.3	3.5	1.9	6.6	2.89	0.75	11.2		0.30	0.42	
	Non‐pregnant mare	38.9	33.9	44.2	50.6	40.1	61.1	35.2	29.6	41.2	2.48	1.37	4.50		0.54	0.16	
	Stallion	5.3	3.4	8.2	7.2	3.4	14.9	4.7	2.7	8.0	2.0	0.59	6.79		0.54	0.38	
**Sex**	Male	56.9	51.7	62.2	43.4	32.7	54.0	61.3	55.4	67.3	Ref			.003	Ref		.002
	Female	43.1	37.8	48.3	56.6	46.0	67.3	38.7	32.7	44.6	2.31	1.32	4.04		0.47	0.15	
**Pregnant**	No	95.9	93.8	98.0	94.0	91.6	96.6	96.5	94.2	98.7	Ref			.4	Ref		.9
	Yes	4.1	2.0	6.2	6.0	3.4	8.4	3.5	1.3	5.8	1.71	0.46	6.26		‐0.05	0.42	
**Breed**	Welsh A	21.5	17.2	25.9	43.4	32.7	54.0	14.5	10.1	18.8	Ref			<.001	Ref		<.001
	Welsh B and C	12.1	8.6	15.6	12.0	5.0	19.1	12.1	8.1	16.1	0.34	0.14	0.81		−0.75	0.24	
	Welsh D	11.2	7.9	14.6	9.6	3.3	16.0	11.7	7.8	15.7	0.27	0.11	0.69		−0.86	0.25	
	Cob	25.1	20.5	29.7	8.4	2.5	14.4	30.5	24.8	36.1	0.09	0.04	0.24		−1.80	0.20	
	Connemara	13.3	9.7	16.9	4.8	0.2	9.4	16.0	11.5	20.5	0.1	0.03	0.32		−1.49	0.26	
	Others^a^	16.8	12.8	20.8	21.7	12.8	30.6	15.2	10.8	19.6	0.48	0.23	1.02		−0.73	0.26	
**Visit season binary**	Dec ‐ May	31.9	26.9	36.8	31.3	21.3	41.3	32.0	26.3	37.7	Ref			.8	Ref		.4
	June‐November	68.1	63.2	73.1	68.7	58.7	78.7	68.0	62.3	73.7	1.11	0.55	2.27		0.21	0.24	
**Visit Season**	Mar – May	15.9	12.4	20.2	16.9	10.3	26.3	15.6	11.7	20.6	Ref			.8			.5
	Jun – Aug	33.9	29.1	39.1	38.6	28.8	49.3	32.4	27.0	38.4	0.88	0.33	2.33		0.41	0.34	
	Sep – Nov	34.2	29.4	39.4	30.1	21.3	40.7	35.5	30.0	41.6	0.73	0.33	1.62		0.10	0.35	
	Dec ‐ Feb	15.9	12.4	20.2	14.5	8.5	23.6	16.4	12.4	21.4	0.68	0.25	1.84		0.12	0.40	
**Establishment Type**	Stud	23.9	19.4	28.4	32.5	22.5	42.6	21.1	16.1	26.1	Ref			.3	Ref		.07
	Livery yard	23.0	18.5	27.5	21.7	12.8	30.6	23.4	18.2	28.6	0.61	0.25	1.46		‐0.23	0.29	
	Riding school	53.1	47.8	58.4	45.8	35.1	56.5	55.5	49.4	61.6	0.55	0.26	1.18		‐0.58	0.26	
**Time to consume meal (min)**	n/a	11 (8 – 15)			11 (8‐17)			11 (8‐14))			1.0	0.97	1.03	.9	0.004	0.01	.7
**Percentage of meal consumed**	n/a	100 (100‐100)			100 (100‐ 100)			100 (100 – 100)			1.0	0.98	1.04	.6	‐0.02	0.02	.4
**Main activity**	Show, breed, companion, pet	29.5	24.6	34.4	43.4	32.7	54.0	25.0	19.7	30.3	Ref			.005	Ref		<.001
	Riding or driving	70.5	65.6	75.4	56.6	46.0	67.3	75.0	69.7	80.3	0.41	0.22	0.77		‐0.77	0.19	
**Exercise score** ^b^	n/a	11.4 (1.5 – 18.9)			7.6 (0 – 15.6)			13.0 (2.3 – 20)			0.97	0.94	1.0	.03	‐0.04	0.01	<.001
**Weight status**	Under/normal (BCS 1‐6)	24.8	20.2	29.4	15.7	7.8	23.5	27.7	22.3	33.2	Ref			.03	Ref		.004
	Overweight (BCS 7‐9)	75.2	70.6	79.8	84.3	76.5	92.2	72.3	66.8	77.7	2.20	1.07	4.50		0.47	0.16	
**Cresty Neck Score**	1 and 2	34.8	29.7	39.9	20.5	11.8	29.2	39.5	33.5	45.4	Ref			<.001	Ref		<.001
	3	40.1	34.9	45.3	30.1	20.3	40.0	43.4	37.3	49.4	1.27	0.62	2.59		0.19	0.16	
	4 and 5	25.1	20.5	29.7	49.4	38.6	60.2	17.2	12.6	21.8	5.45	2.68	11.09		0.82	0.18	
**Hours/day at pasture summer**	0‐6	19.5	15.3	23.7	30.1	20.3	40.0	16	11.5	20.5	Ref			.02	Ref		<.001
	7‐12	22.4	18.0	26.9	20.5	11.8	29.2	23	17.9	28.2	0.40	0.16	0.99		‐0.52	0.28	
	12‐24	58.1	52.9	63.4	49.4	38.6	60.2	60.9	55.0	66.9	0.35	0.17	0.74		‐0.95	0.23	
**Hours/day at pasture winter**	0	15.0	11.2	18.8	18.1	9.8	26.4	14.1	9.8	18.3	Ref			.4	Ref		.8
	1‐6	30.1	25.2	35.0	33.7	23.6	43.9	28.9	23.4	34.5	1.05	0.42	2.64		−0.18	0.31	
	7‐18	26.8	22.1	31.6	18.1	9.8	26.4	29.7	24.1	35.3	0.50	0.18	1.38		−0.22	0.33	
	19‐24	28.0	23.2	32.8	30.1	20.3	40.0	27.3	21.9	32.8	0.94	0.38	2.37		−0.28	0.31	
**Forage currently fed**	None	5.9	3.4	8.4	2.4	0	5.7	7.0	3.9	10.2	0.31	0.05	1.77	.02	−0.46	0.39	.03
	Hay	38.6	33.5	43.8	37.3	26.9	47.8	39.2	33.1	45.0	Ref				Ref		
	Haylage	51.6	46.3	56.9	50.6	39.8	61.4	52.0	45.8	58.1	0.98	0.50	1.90		−0.02	0.20	
	Soaked hay	3.8	1.8	5.9	9.6	3.3	16.0	2.0	0.3	3.6	5.88	1.63	21.14		0.98	0.37	
**Forage weighed (n = 319)**	No	90.6	87.4	93.8	90.1	84.0	96.7	90.8	87.1	94.2	Ref			1.0	Ref		.6
	Yes	9.4	6.2	12.6	9.9	3.3	16.0	9.2	5.8	12.9	0.99	0.34	2.86		0.21	0.36	
**Number of additional feeds**	n/a	1 (0‐2)			1 (0‐1)			1 (0‐2)			0.82	0.60	1.12	.2	‐0.14	0.10	.1
**Rug used in Summer**	No	70.5	65.6	75.4	68.7	58.7	78.7	71.1	65.5	76.6	Ref			.9	Ref		.4
	Yes	29.5	24.6	34.4	31.3	21.3	41.3	28.9	23.4	34.5	1.06	0.56	2.01		0.14	0.19	
**Rug used winter**	No	27.4	22.7	32.2	33.7	23.6	43.9	25.4	20.1	30.7	Ref			.2	Ref		.02
	Yes	72.6	67.8	77.3	66.3	56.1	76.4	74.6	69.3	79.9	0.65	0.35	1.24		‐0.47	0.19	

^a^ Others: Fell (3.8%), Shetland (3.8%), New Forest (2.9%), Highland (2.1%), Dartmoor (1.8%), Dales (1.5%), and Exmoor (0.9%).

^b^ Hours exercised per week walking, trotting, cantering, jumping or galloping were multiplied by 1, 2, 4, 5 or 6, respectively, and the sum calculated as overall exercise score.

**Table 2:** Descriptive data for clinical manifestations of EMS in all (n = 339), EMS positive (n = 83) and EMS negative (n = 256) ponies; univariable, multilevel logistic regression of clinical manifestations associated with EMS diagnosis (n = 339) and univariable, multilevel linear regression of clinical manifestations associated with Log_e_ (insulin_120_ + 1) (n = 320) in native ponies and cobs in northwest England and north Wales. Categorical variables are presented as percentages with upper and lower 95% CI and continuous variables as median (IQR)

Variable	Category	All ponies (n = 339) (%)	Lower 95% CI	Upper 95% CI	EMS positive (n = 83) (%)	Lower 95% CI	Upper 95% CI	EMS negative (n = 256) (%)	Lower 95% CI	Upper 95% CI	Binary outcome Odds Ratio (95% CI)	Lower 95% CI	Upper 95% CI	*P*‐Value	Continuous outcome Coefficient (95% CI)	S.E.	*P*‐Value
Laminitis in the last 5 years	No	90.3	87.1	93.4	69.9	60.0	79.7	96.9	94.7	99.0	Ref			<.001	Ref		<.001
	Yes	9.7	6.6	12.9	30.1	20.3	40.0	3.1	1.0	5.3	14.40	5.87	35.28		1.68	0.23	
Growth ring score	1	14.5	10.7	18.2	7.2	1.7	12.8	16.8	12.2	21.4	Ref			.001	Ref		.009
	2	44.2	39.0	49.5	33.7	23.6	43.9	47.7	41.5	53.8	1.78	0.64	4.99		0.38	0.21	
	3	41.3	36.1	46.5	59.0	48.5	69.6	35.5	29.7	41.4	4.80	1.62	12.40		0.67	0.23	
Hoof pastern axis	Straight	76.1	71.6	80.6	71.1	61.3	80.8	77.7	72.6	82.8	Ref			.2	Ref		.5
	Broken forward	13.3	9.7	16.9	13.3	6.0	20.5	13.3	9.1	17.4	1.26	0.57	2.80		0.087	0.21	
	Broken back	10.6	7.3	13.9	15.7	7.8	23.5	9.0	5.5	12.5	2.04	0.92	4.53		0.26	0.23	
Supraorbital fat score	1	18.3	14.2	22.4	13.3	6.0	20.5	19.9	15.0	24.8	Ref			.004	Ref		.01
	2	49.0	43.6	54.3	37.3	26.9	47.8	52.7	46.6	58.9	1.01	0.48	2.15		0.19	0.19	
	3	32.7	27.7	37.7	49.4	38.6	60.2	27.3	21.9	32.8	2.45	1.15	5.26		0.58	0.21	
Blood glucose (t = 0) (mmol/L)	n/a	5.0 (4.6 – 5.4)			5.1 (4.6 – 5.4)			5.0 (4.6 – 5.4)			1.11	0.72	1.73	.6	0.053	0.12	.7
Change in Blood glucose (mmol/L)	n/a	3.0 (1.6 – 5.0)			4.6 (3.2 – 6.0)			2.5 (1.3 – 4.2)			1.51	1.29	1.75	<.001	0.27	0.03	<.001
Insulin (t = 0 min) (µIU/mL)	n/a	0.0 (0.0 – 3.8)			6.4 (2.0 – 15.4)			0.0 (0.0 – 1.3)									
Log_e_ (Insulin_0_ + 1)	n/a	0.0 (0.0 – 1.6)			2.0 (1.1 – 2.8)			0.0 (0.0 – 0.8)							0.048	0.006	<.001
Insulin (t = 120 min) (µIU/mL)	n/a	45.7 (18.7 – 124.0)			292.2 (180.8 – 477.0)			30.5 (15.3 – 55.8)									
Log_e_ (Insulin_120_ + 1)	n/a	3.9 (3.0 – 4.8)			5.7 (5.2 – 6.2)			3.4 (2.8 – 4.0)									

Due to the nature of sampling, data were clustered within horse yards (level two units) therefore multilevel (horse, yard) univariable logistic and linear regression models were used to identify explanatory factors associated with a binary outcome of EMS status (positive or negative) and a continuous outcome of Log_e_ (insulin_120_ + 1), respectively. Within‐yard clustering was accounted for by incorporation of yard as a random intercept terms in all models.^22^

The prevalence of EMS was estimated after adjusting for clustering of animals within yard using the constant parameter estimate (*β_0_*) and the standard errors derived from an intercept‐only multilevel model incorporating yard as a random effect.

Potential correlations between explanatory variables were assessed using Pearson or Spearman coefficient according to the type and distribution of data. Correlated variables (correlation coefficient > 0.7) were excluded or modified. Variables with a *P*‐value < .25 were entered into multilevel, multivariable models.^23^ Variables with the greatest *P‐*value were sequentially removed in a step‐wise backwards elimination procedure with concurrent assessment for confounding, until all variables in the final model had *P‐*values < .05. Eliminated variables were individually inserted back into the multivariable model to check if they improved the fit of the final model. Effect modifications were tested for clinically plausible interaction terms such as age*main activity and visit season*hours/day pasture in summer.

Associations between clinical manifestation variables and binary (EMS diagnosis) and continuous outcome (Log_e_ (insulin_120_ + 1)) were assessed using multilevel (horse, yard) univariable logistic and linear regression, respectively. Data were analysed using commercial software MLwiN (version 3.01) (Centre for Multilevel Modelling, University of Bristol) and SPSS (version 24) (IBM Corp.), and significance was assumed at *P* < .05.

## 3 RESULTS

### 3.1 Study population

A total of 354 animals were examined at 64 properties. Of the establishments invited to participate, 19/151 studs, 19/154 livery yards and 26/61 riding schools were enrolled in the study, resulting in an overall establishment response rate of 17.5%. Median (range) animals included per establishment was 5 (1‐12). Visits were conducted year‐round from May 2015 to November 2016. Animals from which a second blood sample could not be obtained (n = 2), and any positive for PPID (n = 4) were excluded. Nine (2.6%) animals consumed <50% of feed and 19 animals (5.5%) consumed 50%‐75% of feed, resulting in sample sizes of n = 339 for the binary outcome (EMS positive/negative) and n = 320 for the continuous outcome (log_e_ (insulin_120_ + 1)).

In the binary outcome data set (n = 339) median (IQR) age was 9 (6‐11) years and 51.6% were geldings, 5.3% were stallions and 43.1% were mares, of which 9.6% were pregnant. Pure‐ or crossbred Welsh (44.8%), Connemara (13.3%), Fell (3.8%), Shetland (3.8%), New Forest (2.9%), Highland (2.1%), Dartmoor (1.8%), Dales (1.5%) and Exmoor (0.9%) ponies, and cobs (25.1%) were included. Most animals (75.2%) were overweight (BCS 7‐9), with the remainder (24.8%) ideal weight (BCS 4‐6). Further descriptive data and univariable analysis are presented in Tables 1 and 2.

### 3.2 Binary outcome (EMS diagnosis)

Eighty‐three animals of 339 were classified as positive for EMS. The overall prevalence of EMS adjusted for clustering within yard was 23.3% (95%CI 17.9%–29.8%). In 9/339 (2.7%) animals insulin_0_ was > 33 µIU/mL; eight of these were also categorised as EMS positive based on insulin_120_. Median (IQR) insulin_120_ was 292 (181–477) µIU/mL in EMS positive animals and 30.5 (15.3–55.8) µIU/mL in EMS negative animals (Table 2).

#### 3.2.1 Risk factors

Univariable, multilevel logistic regression for risk factors (Table 1) resulted in 10 explanatory variables being entered a multivariable model, of which six were retained in the final model (Table 3). No clinically plausible, significant interaction terms were identified. The odds of a positive diagnosis of EMS increased by 1.38 (95% CI 1.24–1.54) with each year increase in a pony's age. Female animals and those involved in more sedentary main activities including showing, breeding and being companions had increased odds of being positive for EMS compared to those ridden or driven. Compared to the Welsh section A, the other Welsh, Connemara and cob breeds all had decreased odds of EMS. Furthermore, animals with a BCS ≥ 7/9 and those that were turned out to pasture for shorter periods during the summer had increased odds of being positive for EMS.

**Table 3:** Multivariable, multilevel logistic (n = 339) and linear (n = 320) regression models of risk factors associated with EMS diagnosis and Log_e_ (insulin_120_ + 1), respectively, in native ponies and cobs in northwest England and north Wales

Variable	Category	Logistic regression (n = 339)				Linear regression (n = 320)		
		OR	Lower 95% CI	Upper 95% CI	*P* ‐value	Coefficient	S.E.	*P*‐value
Age	Years	1.38	1.24	1.54	<.001	0.13	0.02	<.001
Sex	Male	Ref			.02	Ref		.02
	Female	2.13	1.13	4.01		0.27	0.12	
Breed	Welsh A	Ref			<.001	Ref		<.001
	Welsh B and C	0.35	0.13	0.93		−0.60	0.21	
	Welsh D	0.15	0.05	0.45		−0.79	0.22	
	Cob	0.14	0.05	0.39		−1.35	0.18	
	Connemara	0.06	0.02	0.20		−1.48	0.22	
	Others	0.51	0.21	1.20		−0.58	0.19	
Main activity	Riding/ driving	Ref			<.001	Ref		<.001
	Showing/breeding/companion/other	3.51	1.75	7.04		0.66	0.16	
Time at grass in summer (hours)	0‐6	Ref			<.001	Ref		<.001
	7 – 12	0.25	0.10	0.64		‐0.46	0.21	
	>12	0.21	0.10	0.47		‐0.91	0.17	
Overweight (BCS ≥ 7/9)	No	Ref			.003	Ref		<.001
	Yes	3.48	1.53	7.92		0.48	0.13	
Winter rugging	No				n/s	Ref		.02
	Yes					‐0.36	0.16	
Within‐yard variance (S.E.)		0.000 (0.000)				0.139 (0.059)		

#### 3.2.2 Clinical manifestations

Univariable, multilevel logistic regression (Table 2) showed that animals reported to have had at least one episode of laminitis during the last 5 years were 14.4 (95% CI 5.9–35.3) times more likely to be positive for EMS than those with no recent history of laminitis. Growth ring and supraorbital fat scores of 3/3 were both associated with increased odds of EMS compared to scores of 1/3. A greater increase in blood glucose concentration (glucose_120_–glucose_0_) was associated with increased odds of EMS, however, glucose_0_ was not.

### 3.3 Continuous Outcome (Log_e_ (insulin_120_ + 1), n = 320)

**Figure 1:**
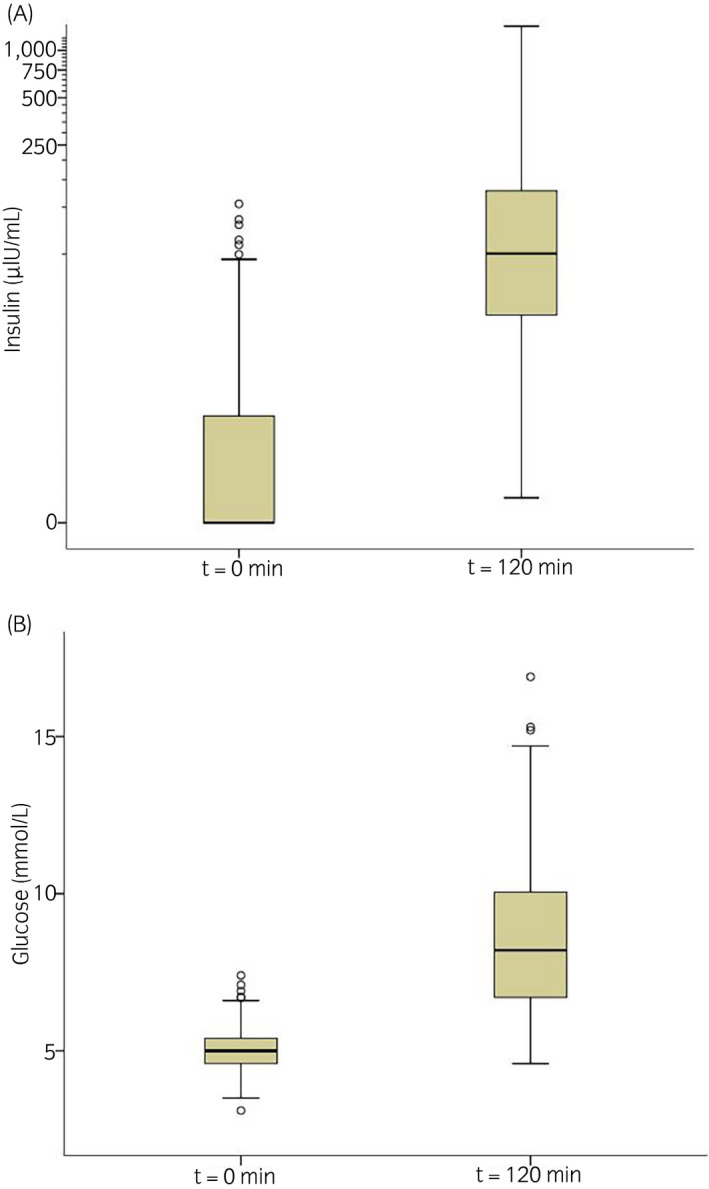
Box and whisker plot of serum insulin (A) and blood glucose (B) concentrations after fasting (t = 0 min) and 120 minutes following 1 g/kg glucose (t = 120 min) in 320 ponies in northwest England and north Wales

Median (IQR) insulin_120_ for all animals was 50.3 (19.9–127.8) µIU/mL (Figure 1).

#### 3.3.1 Risk factors

Univariable, multilevel linear regression (Table 1) resulted in 10 explanatory variables being entered into a multivariable model. Seven factors were retained in the final model (Table 3). In addition to the six variables identified by binary logistic regression, winter rugging was associated with decreased Log_e_ (insulin_120_ + 1); with an estimated coefficient (s.e.) of −0.36 (0.16).

#### 3.3.2 Clinical manifestations

Univariable, multilevel linear regression is provided in Table 2. The same clinical manifestations were associated with increased Log_e_ (insulin_120_ + 1) as were associated with a positive EMS diagnosis.

## 4 DISCUSSION

This study represents the first robust prevalence estimate of EMS in susceptible UK breeds using a dynamic, oral test for ID. The prevalence of 23.3% demonstrates that a significant proportion of 3‐14‐year‐old native ponies and cobs in the UK are at increased risk of developing laminitis. After allowing for fixed effects in the multivariable models there was minimal remaining within‐yard clustering with estimates of zero for the binary model and 0.14 for the continuous model (Table 3), suggesting the fixed effects such as breed and main activity account for much of the yard‐level variation. Studies in Australia on mixed breed ponies^10^ and in the USA on light breed horses^13^ estimated the prevalence of EMS to be 27% and 18%, respectively, based on a basal insulin of >20 µIU/mL. Applying the same diagnostic criteria in the current study would have resulted in a much lower prevalence of EMS (5.7%), likely reflecting differences in the period of fasting and the insulin assay used, as well as between the populations studied.

Several diagnostic cut‐offs for basal fasting and post‐OGT insulin concentrations for EMS have been published,^2^, ^24^ and in a recent study, thresholds were calculated that quantified laminitis risk in ponies subsequently fed a high carbohydrate diet.^25^ In our study, the cut‐off was taken from a recent consensus document^1^ using an equivalent assay. Different cut‐offs may have resulted in a different prevalence and possibly associated risk factors for a positive diagnosis, but would not have affected the risk factors associated with insulin_120_. Although EMS is defined as collection of historical, clinical and laboratory risk factors for endocrinopathic laminitis, ID is the central and consistent feature,^1^ and increasingly the terms ID and EMS are being used interchangeably. Because of this, in the current study a diagnostic test for ID was used to determine EMS status. In clinical practice a diagnosis of EMS is primarily used to identify horses at increased risk of potentially life‐threatening laminitis. This, and the fact that management of EMS is centred on low‐risk and inexpensive modification of diet and exercise means that the false negative rate should be minimised by using cut‐offs which prioritise sensitivity over specificity. It is important to consider the insulin assay being used, as results can vary considerably.^17^

Equine metabolic syndrome increases risk for endocrinopathic laminitis, so the prevalence of EMS and laminitis are related but not directly comparable. In this study 9.7% of all ponies were reported to have had at least one episode of laminitis in the previous 5 years, and of those, 76% were EMS positive. Veterinary advice was sought in 56% of the reported episodes of laminitis. The frequency of previous laminitis may be an underestimate, as the median (IQR) duration of ownership of all animals was 3 (1‐6) years. An episode of laminitis during the previous 5 years was strongly associated with an increased risk of current EMS, indicating that animals are at greater risk of laminitis if they have had an episode in the last 5 years. Other estimates of the frequency of endocrinopathic laminitis in equids vary considerably, ranging from 1.5% to 34%.^8^ In most studies laminitis is defined by foot lameness as detected by the owner. Divergent hoof growth rings have been shown previously to occur in feet with histological evidence of laminitis,^26^ and the association between hoof ring score and EMS and insulin_120_ shown in this study further supports this. The use of hoof morphology as a simple method of identifying animals at risk of EMS is poorly described and requires further research.

Several risk factors associated with increased insulin_120_ and a diagnosis of EMS support findings from previous studies. Increasing age was identified as a risk factor for EMS, supporting the association between age and ID that has been recognised in humans and horses.^10^, ^27^ This study also supports the association between adiposity and EMS, with both obesity (BCS ≥ 7/9) and CNS increasing the odds of EMS in the univariable analysis, and obesity remaining in both multivariable models. CNS was excluded from the multivariable model only because of a strong correlation with obesity. The causal relationship between obesity and EMS remains unclear, but a proinflammatory state^28^ and dysregulation of adipokines, for example adiponectin and leptin^3^, ^13^ are likely. An association between BCS and ID has been lacking in some smaller experimental studies,^29^ while others have demonstrated an effect of diet^30^ or regional adiposity.^2^, ^29^ Although BCS is an imperfect predictor of total body fat^31^ these results support its use as a modifiable risk factor and to screen for animals at increased risk of EMS.

This study chose breeds considered at increased risk of EMS, and in which previous UK‐based research is lacking. These results may not be transferrable to other breed groups. This focused approach did allow the study to show variation in risk between the breeds studied, with increased odds of EMS in the smaller Section A compared with the other sections within the Welsh mountain pony breed. Other studies have found an inverse correlation between height and baseline insulin, driven, in part by genetic factors.^32^ Some breed groups including Highland ponies and Shetlands were less well‐represented, potentially limiting the power to detect breed differences in these breeds.

This is the first study to show a sex effect with mares having greater odds of EMS than geldings and stallions. Insulin antagonism by female sex hormones might contribute to this finding.^33^ Although pregnancy has been cited as contributing to ID in people^34^ this is less likely in horses, particularly during the first and second trimesters of pregnancy.^35^ Pregnancy was not found to increase the odds of EMS in the current study although only 14 (4.1%) of the animals were pregnant.

Ponies and cobs with sedentary main activities including showing, breeding and being companions had increased odds of being positive for EMS compared to those ridden or driven. Although the exercise score was significantly associated with EMS and insulin_120_ in the univariable models, it was only the main activity that was retained in the final models. Previous research has been conflicting as to the level of exercise required to improve ID. Moderate intensity exercise has been recommended by consensus,^1^ but even very low levels of exercise have also been shown to affect morphological indicators of obesity,^36^ which may indirectly decrease the risk of EMS. The results of this study support exercise as a modifiable factor than could be used to decrease the odds of EMS.

Absence of rugging in winter and decreased turnout at pasture in the summer were two novel risk factors associated with increased insulin_120_ and risk of EMS identified in this study. These contradict most current opinion,^1^ although these data only estimate association, not causality and it is possible that they represent owners identifying animals at increased risk of EMS (due to obesity or previous laminitis) and modifying management according to current advice of keeping horses unrugged to promote cold‐induced thermogenesis and restricting summer turn out to reduce grass intake. There was no significant association between previous laminitis and winter rugging or summer turnout at pasture identified on chi‐squared analysis (data not shown). The rationale for leaving a horse unrugged is dependent on the ambient temperature being lower than the animal's lower critical temperature (LCT), meaning that additional energy is used to maintain core body temperature. Estimates of the horse's LCT vary between 0‐5°C,^37^ but horses will adapt to their environment. Mean minimum daily air temperature during January across the region studied is typically 2°C,^38^ close to the LCT. It is also possible that rugging a horse in cold weather might increase movement and hence exercise. Clipping the coat is probably more common in horses engaged in strenuous activities, and means they are more likely to be rugged. Although an exercise score and main activity were included in the analyses, clipping is a possible confounding factor in the observed association between absence of rugging and EMS. This warrants further research.

A limitation and source of potential bias in this study was the incomplete consumption of glucose by some animals. The OGT was selected for diagnosis of EMS as it is a dynamic test that assesses the enteroinsular axis as well as insulin resistance and is practical to use in the field. It has reasonable repeatability^20^ and has been shown to predict laminitis risk in ponies fed a high carbohydrate diet.^25^ All animals consuming >75% of glucose were classified together for EMS diagnosis cut‐off and included in the analysis for the outcome of insulin_120_. A previous study^20^ unexpectedly found that insulin responses were greater following 0.75 g/kg compared to 1 g/kg glucose dose. Intestinal absorption of glucose might be saturated at doses >0.75 g/kg, and the authors in the study above speculated that differences in consumption rate, or other gastrointestinal factors might have contributed. In the current study the exclusion of animals consuming <75% and <50% of the glucose from the insulin_120_ and EMS diagnosis outcomes, respectively, might have introduced bias or imprecise prevalence estimates. Willingness to voluntarily consume an unfamiliar feed might be associated with obesity and ID, overestimating the prevalence of EMS. Additionally, owners of obese or previously laminitic animals might have been more likely to volunteer, introducing selection bias.

## 5 CONCLUSION

In conclusion, this study shows that there is a high prevalence of EMS and increased risk of laminitis in native ponies and cobs aged 3‐14 years in a specific region of the UK. Several historical, management and morphological risk factors are identified that owners and veterinarians can use to identify animals that are at increased risk of EMS, allowing more accurate selection of animals for management based on a presumptive diagnosis, or for further diagnostic testing. It also highlights modifiable risk factors to assist in the management of EMS. Further prospective studies are warranted to determine whether novel associated risk factors such as decreased summer grazing are causal, or a result of reactive management changes by owners.

## ETHICAL ANIMAL RESEARCH

This study was approved by the veterinary research ethics committee of the University of Liverpool, UK, project VREC314.

## CONFLICT OF INTERESTS

No competing interests have been declared.

## AUTHOR CONTRIBUTIONS

The study was designed by H. Carslake, G. Pinchbeck and C. McGowan, and executed by H. Carslake. All authors contributed to data analysis and interpretation and preparation and approval of the final manuscript.

## OWNER INFORMED CONSENT

Owner informed consent was obtained for all animals prior to enrolment in the study.

### Peer Review

The peer review history for this article is available at https://publons.com/publon/10.1111/evj.13378.

## Supporting information

Supplementary Material

Supplementary Material

Supplementary Material

Supplementary Material

## Data Availability

The data that support the findings of this study are available from the author upon reasonable request.
