# Supplementary material for: Equine metabolic syndrome in UK native ponies and cobs is highly prevalent with modifiable risk factors
Source: Equine Vet J. 2020 Dec 3;53(5):923–34. doi: 10.1111/evj.13378 (PMC8451835; doi:10.1111/evj.13378)
Supplement: Supplementary file 1 — Supplementary Material [file EVJ-53-923-s002.pdf]

Equine Veterinary Journal BEVA

Supplementary Item 1

Grading Scheme for hoof growth rings and supraorbital fat.

Hoof growth ring divergence score

Grade	Description	Example
1	All growth rings are parallel to the coronary band, or show only very mild separation towards the heel.	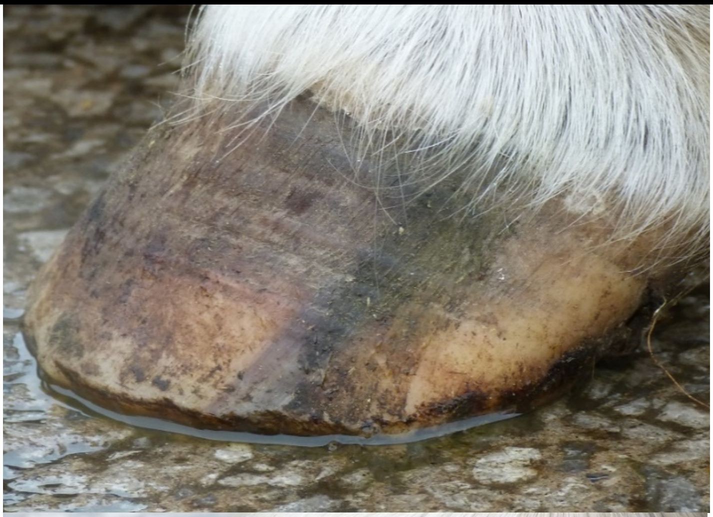
2	Toward the heel, growth rings show moderate separation but remain straight and/or show mild distal deviation	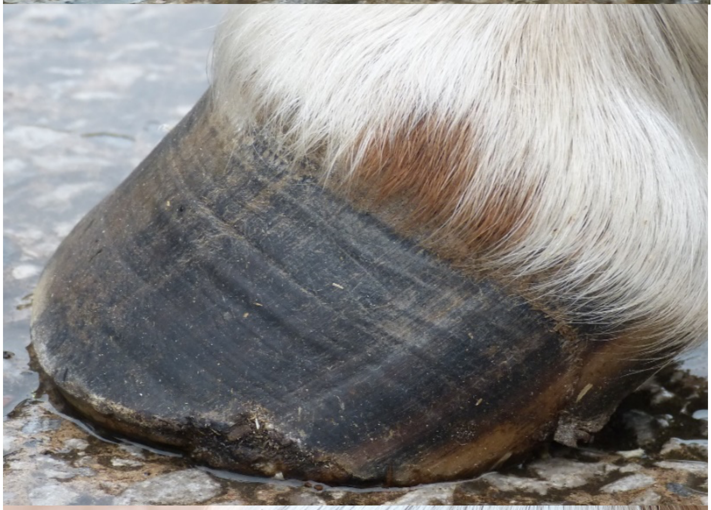
3	Towards the heel, growth rings show marked separation and/or marked distal deviation	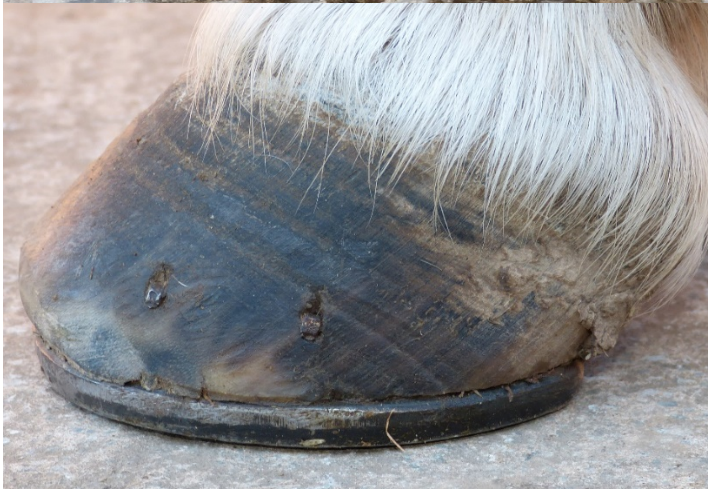

Hoof growth ring prominence score

Hooves should be assessed away from any areas of rasping that may have occurred during trimming or farriery

Grade	Description	Example
1	Hoof capsule is smooth, or growth rings only just visible or palpable	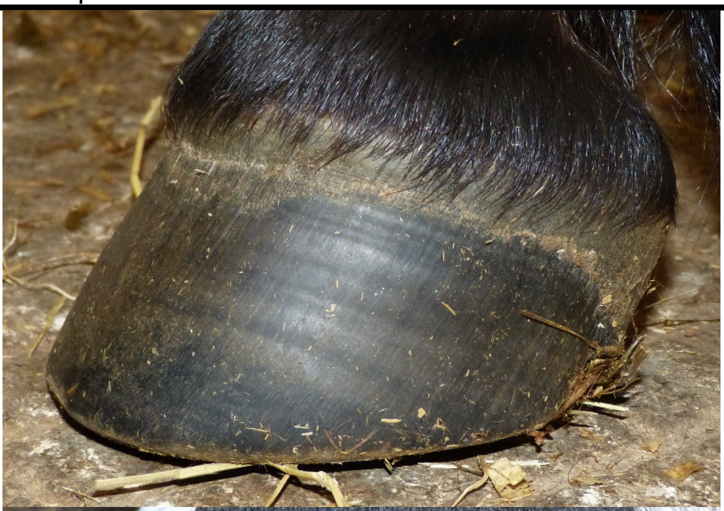
2	Moderate depth growth rings clearly visible and palpable	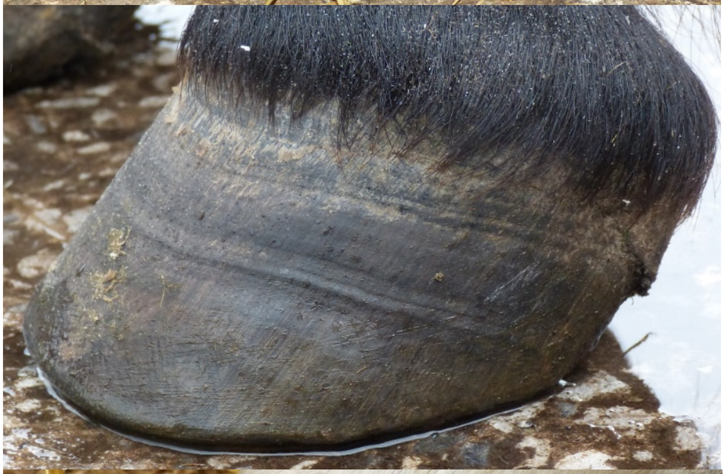
3	Prominent, deep growth rings visible	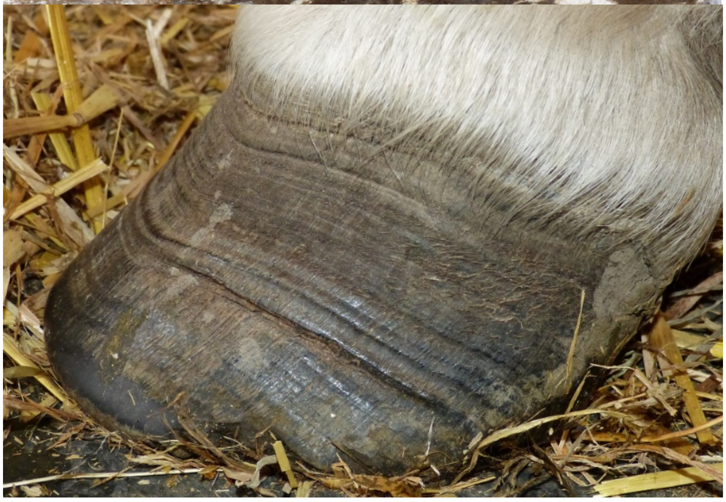

Supraorbital fat score

Grade 1: The soft tissue within the supraorbital fossa is concave relative to the surrounding bone.

Grade 2: The soft tissue within the supraorbital fossa is level with the surrounding bone.

Grade 3: The soft tissue within the supraorbital fossa is convex relative to the surround bone.
