# Supplementary material for: Equine metabolic syndrome in UK native ponies and cobs is highly prevalent with modifiable risk factors
Source: Equine Vet J. 2020 Dec 3;53(5):923–34. doi: 10.1111/evj.13378 (PMC8451835; doi:10.1111/evj.13378)
Supplement: Supplementary file 2 — Supplementary Material [file EVJ-53-923-s004.pdf]

Equine Veterinary Journal BEVA

Supplementary Item 2: Correlation and linear regression from paired analyses of serum insulin concentration in 39 samples on the Immulite 2000XPi and the Immulite 2000 chemiluminescent analysers. Statistical analysis performed using SPSS 24[1].

Equine serum samples (n=39) were analysed on two models of a chemiluminescent analyser (Immulite 2000[2] and Immulite 2000XPi[2]). Descriptive data are shown in Table 1.

Assay	Median (IQR)
Immulite 2000	33.45 (61.75)
Immulite 2000XPi	59.50 (103.25)

Table 1: Descriptive data (median (IQR) for paired analysis of equine serum samples (n=39) on 2 models of a chemiluminescent analyser (Immulite 2000 and Immulite 2000XPi).

Assessment of a scatter plot (Fig. 1) was suggestive of a strong linear relationship between the two variables, with a positively skewed distribution. The distribution of the serum insulin concentration in both data sets was significantly skewed (Shapiro-Wilk test for normality *P* <0.001, Fig. 2), and Pearson and Spearman's Rank coefficients show a very strong positive correlation of 0.998 and 0.996, respectively (*P* <0.001).

Figure 1

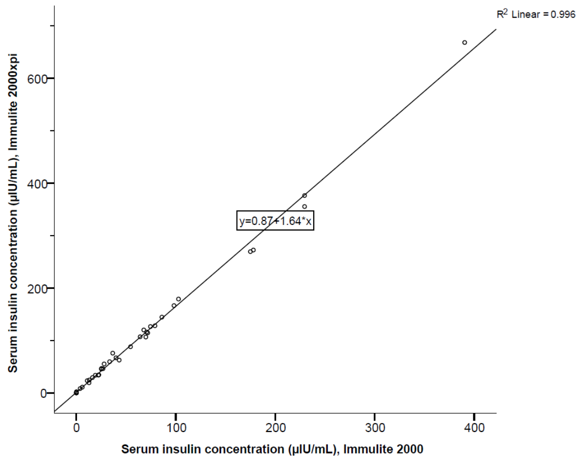

Fig. 1 Scatter plot of serum insulin concentration (µIU/mL) with line of best fit from paired analysis with two chemiluminescent analysers (Immulite 2000 (*x*-axis) and Immulite 2000XPi (*y*-axis))

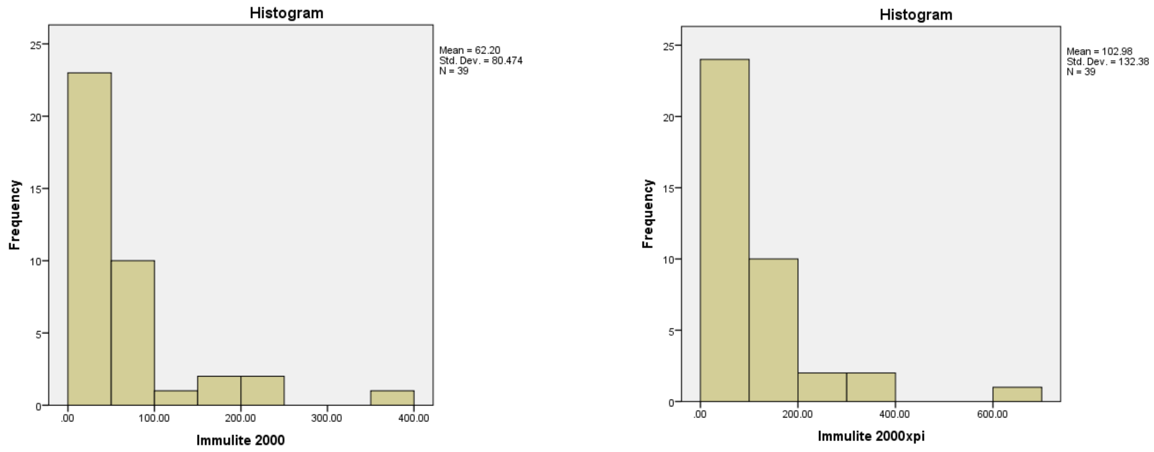

A B

Fig. 2 Histograms showing distribution of serum insulin concentration on two chemiluminscent analysers (Immulite 2000, A and Immulite 2000XPi, B)

Linear Regression

Simple regression analysis was performed to transform serum insulin concentrations ([insulin]) using the older Immulite 2000 analyser as the independent variable ($[\text{insulin}]_{2000XPi}$) and the newer Immulite 2000XPi as the dependent variable ($[\text{insulin}]_{2000}$). Given the very strong correlation, (r = 0.998, R^2 = 0.996) linear regression was used. The intercept was 0.87 and gradient (95% CI) 1.64 (1.61 – 1.68), resulting in the regression model:

$$[\text{insulin}]_{2000XPi} = 0.87 + 1.64([\text{insulin}]_{2000}) \qquad (P < 0.001)$$

Assessment of fit

R squared value was 0.996, so 99.6% of the variation in $[\text{insulin}]_{2000XPi}$ can be explained by the model containing $[\text{insulin}]_{2000}$. Residuals were approximately normally distributed (Fig. 3). A scatter plot of the standardised predicted values versus standardised residuals (Fig. 4) showed heteroscedasticity.

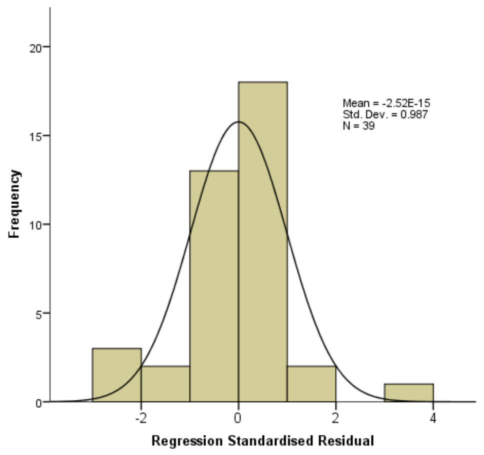

Fig. 3 Histogram of the frequency of residuals of the linear regression model showing an approximately normal distribution

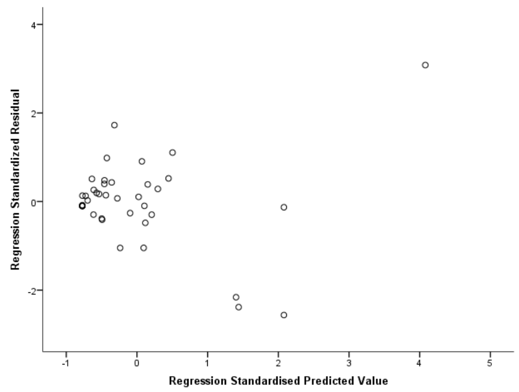

Fig. 4. Scatterplot of residuals against standardised predicted value showing heteroscedasticity.

[1]IBM Corp. New York, USA.

[2]Siemens, Healthcare, Camberley, Surrey, UK.
