# Supplementary material for: Equine metabolic syndrome in UK native ponies and cobs is highly prevalent with modifiable risk factors
Source: Equine Vet J. 2020 Dec 3;53(5):923–34. doi: 10.1111/evj.13378 (PMC8451835; doi:10.1111/evj.13378)
Supplement: Supplementary file 3 — Supplementary Material [file EVJ-53-923-s001.pdf]

Equine Veterinary Journal BEVA

Supplementary Item 3: Questionnaire used for owner interviews.

Prevalence and risk factors for Equine Metabolic syndrome

Client Questionnaire interview schedule

Name of owner: ____________________________ **Date:** _____________________________

A. Signalment/use

1. **Name of pony** ___________________
2. **Breed?** ___________________
3. **Colour?** ___________________
4. **Age?** ___________________ years
5. **Sex?** STALLION / MARE / GELDING
6. **For how many years have you had the pony?** ______________ years
7. **What activities do you do currently with your pony?**
   a. SHOWING / BREEDING / RIDING / DRIVING / COMPANION / PET / OTHER _______
   b. Has your pony had a prior showing/competition career? (YES/NO)
8. **Would you regard your pony as:** VERY THIN / SLIGHTLY UNDERWEIGHT/ IDEAL WEIGHT / SLIGHTLY OVERWEIGHT / VERY OVERWEIGHT?
   a. **Has your pony previously been overweight or very overweight?** (Y / N)
   b. **Is your pony the same weight year round?** (Y / N) (if no _______________________)
9. **Do you monitor your pony's weight?** - weigh tape / weigh bridge? (Y / N)
10. **Do you know the most recent weight of your pony?** __________ On What Date? _________
11. **Is your pony pregnant?** If yes – how many weeks / due date? (exclude third trimester (> 30 weeks) and lactating)
12. **Has your mare had foals in previous years?** 2014 / 2013 / 2012 / 2011 / 2010

    Number of foals before 2010? _______

B. Exercise

1. **How many hours per week does your pony spend in the following activities:-**

Activity	Hours
Schooling - lunged	☐
Schooling - ridden, flat-work	☐
Schooling - ridden, jumping	☐
Hacking	☐
Driving	☐

Other - ____________________________________

2. **Roughly what percentage of the total exercise time each week does your pony spend:-**

Walking	☐	% of total exercise time per week
Trotting	☐	
Cantering	☐	
Galloping	☐	
Jumping	☐	

3. **Overall, what level of work do you consider your pony to be doing?**

☐ light ☐ moderate ☐ intense

C. Feeding/housing

1. **Total number of horses on the premises** ________

2. **How many hours a day is your pony:-**

Turned out at grass:- ☐ hrs/day **in summer**

Turned out at grass:- ☐ hrs/day **in winter**

Turned out to graze-poor paddock (e.g. sand or wood-chip arena): ☐ hrs/day **in summer**

Turned out in graze-poor paddock (e.g. sand or wood-chip arena): ☐ hrs/day **in winter**

Stabled for:- ☐ hours per day in summer

Stabled for:- ☐ hours per day in winter

3. **What Bedding do you use?** Straw / Shavings / Rubber matting / Paper / other / N/A

4. **Do you use winter Rugging?** – Y/N – just at turnout / turnout and stable -

Type:_________________

5. **Do you use Summer Rugging?** – Y/N just at turnout / turnout and stable -

Type:_________________

6. **If turned out to grass, do you ever limit your pony's grazing by use of:-**

Strip-grazing?	Yes ☐	No ☐
Topping?	Yes ☐	No ☐
Grass muzzle?	Yes ☐	No ☐

If YES: ____

7. **Besides grazing, what other food does your pony consume each day & in what quantities?**

Dry HAY ☐Yes ☐No

Amount? __________________ kg / lbs / slices / haynets /other______________

Type of hay? mixed grass / meadow / clover/ ryegrass other _____________

SOAKED HAY Yes ☐ No ☐

Presoaked amount = _______________ kg / lbs / slices / haynets /other______________
How long do you soak the hay? ________________
What temperature is the water? _______________
Other (e.g. steamed) ____________________

Haylage Yes ☐ No ☐
Amount? __________________ kg / lbs / slices / haynets /other______________

Pony nuts/cubes Yes ☐ No ☐
Amount? __________________ kg / lbs / scoops / other __________

Trade name of product/s used_____________________________

Chaff (short chop proprietary feed e.g. HiFi, Alfa A etc.) Yes ☐ No ☐
Amount? __________________ kg / lbs / scoops / other____

Trade name of product/s used……………………………………………………………………………

Coarse mix / Concentrates Yes ☐ No ☐
Amount? __________________ kg / lbs / scoops / other____

Trade name of product/s used……………………………………………………………………………

Other Coarse mix / Concentrates Yes ☐ No ☐

Amount? __________________ kg / lbs / scoops / other ____

Trade name of product/s used……………………………………………………………………………

Balancers? Yes ☐ No ☐

Amount? __________________ kg / lbs / scoops / other __________

Trade name of product/s used……………………………………………………………………………

Vitamin/Mineral supplements

Joint supplements	NAME __________	Amount __________
Vitamins/minerals	NAME __________	Amount __________
Hoof supplements	NAME __________	Amount __________
Electrolytes	NAME __________	Amount __________
Pro/Prebiotics	NAME __________	Amount __________
Herbal supplements	NAME __________	Amount __________
Other (Please specify)	NAME __________	Amount __________

D. Management

1. How do you maintain your pony's feet?

a. TRIMMING or SHOEING / CORRECTIVE TRIMMING (EXPLAIN) / CORRECTIVE FARRIERY (EXPLAIN) ______________________________

b. IN FRONT +/- BEHIND? How often? __________

2. How often are your pony's teeth examined/rasped?

Never / 6 months / 12 months / 24 months / only if there is a problem / never

a. **By who?** - Vet / Lay equine Dentist

E. Medical history

1. Has your horse ever suffered from laminitis? Yes ☐ No ☐

For each episode grade the severity : (**4**:unwilling to walk/lying down; **3**: Quite lame (unwilling to lift up a front leg); **2**: Pottery at walk; **1**: only pottery on corners/Hard surfaces)

	**Episode 1**	**Episode 2**	**Episode 3**	**Episode 4**	**Episode 5**
**Month/Year**					
**Severity**					
**Veterinary advice? (Y/N)**					
**Treatment?**					

Did you make permanent changes to its management after the episode of laminitis?

Yes ☐ No ☐ ***Yes*** *– when and what were they?* ________________________________

2. Have you ever had your pony tested for:

Equine metabolic syndrome? Date: ______ Result: ________

Equine Cushing's disease? Date:______________ Result:_________________

Is your pony on any treatment for this? Yes ☐ No ☐ ______________

3. Has your pony ever had any other medical conditions or lameness diagnosed?

	**Condition 1**	**Condition 2**	**Condition 3**	**Condition 4**
**Month/Year**				
**Diagnosis**				
**Treatment?**				
**Outcome?**				
