# Supplementary material for: Equine metabolic syndrome in UK native ponies and cobs is highly prevalent with modifiable risk factors
Source: Equine Vet J. 2020 Dec 3;53(5):923–34. doi: 10.1111/evj.13378 (PMC8451835; doi:10.1111/evj.13378)

Equine Veterinary Journal BEVA

Supplementary Item 4: Causal web for EMS. Variables included as potentially causal risk factors are plain text, clinical manifestations are underlined.

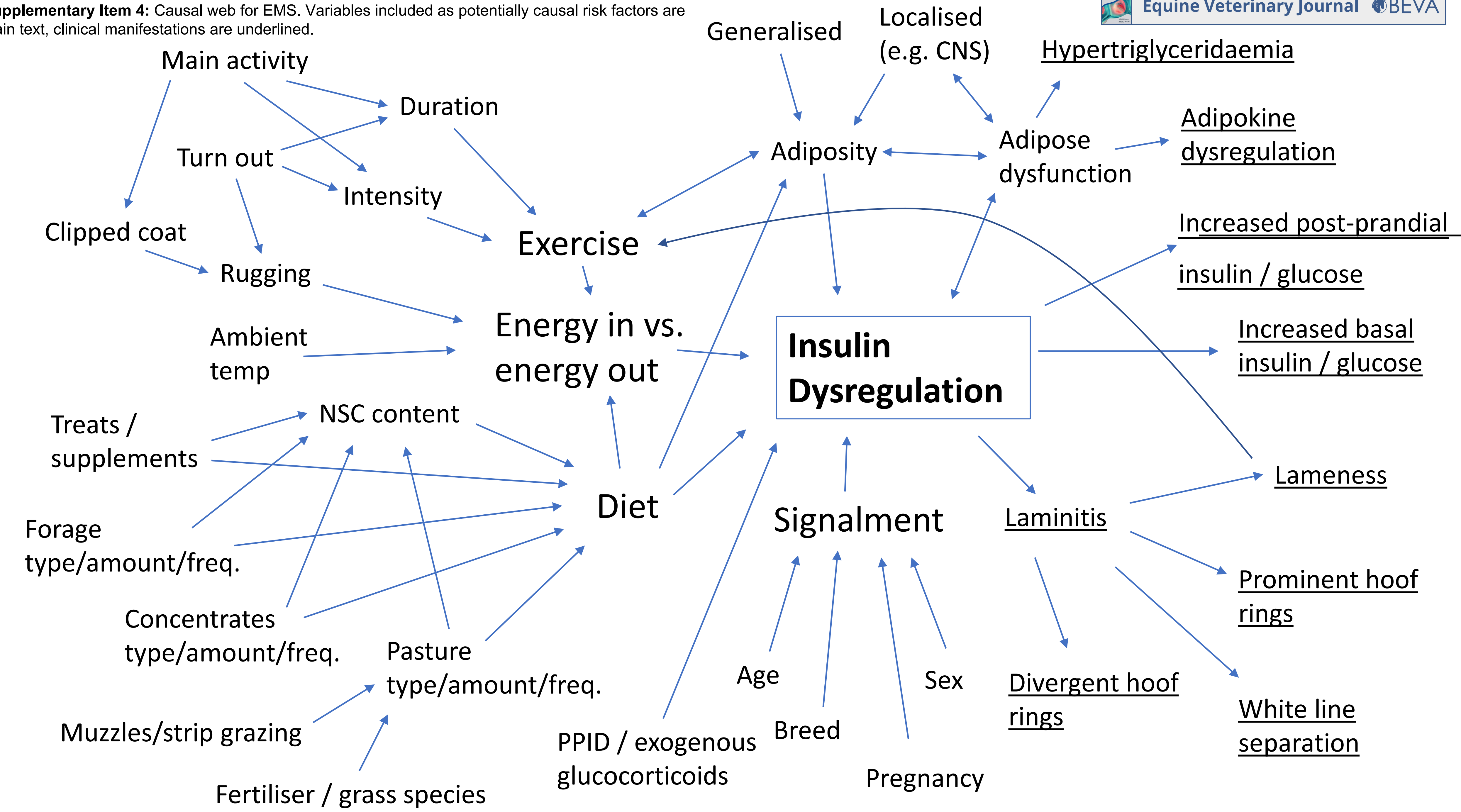

Supplement: Supplementary file 4 — Supplementary Material [file EVJ-53-923-s003.pdf]
